# Supplementary material for: Accuracy comparison of ARIMA and XGBoost forecasting models in predicting the incidence of COVID-19 in Bangladesh
Source: PLOS Glob Public Health. 2022 May 18;2(5):e0000495. doi: 10.1371/journal.pgph.0000495 (PMC10021465; doi:10.1371/journal.pgph.0000495)
Supplement: S1 Text — (DOCX) [file pgph.0000495.s002.docx]

The entire data set was divided into 7 training and 7 test sets for COVID-19 confirmed cases in Bangladesh. It was found nonlinearity and weekly seasonality in each data sets (Fig A). The ARIMA and XGBoost model were built in the training sets and the test sets were used to evaluate the performance of the built ARIMA and XGBoost model. Performing ADF test, it was found that the data was not smooth. Therefore, the Box-Cox transformation was performed to reduce the variation and make the raw data stable [1]. After that the data was decomposed and found weekly seasonality in the data (Fig B) [2]. Taking first order differencing, the data of each training sets found stationary (Fig C). We used auto.arima function under forecast package to fit the ARIMA model for each training set data. The estimated parameters of the fitted ARIMA models for COVID-19 confirmed cases were shown in table A.


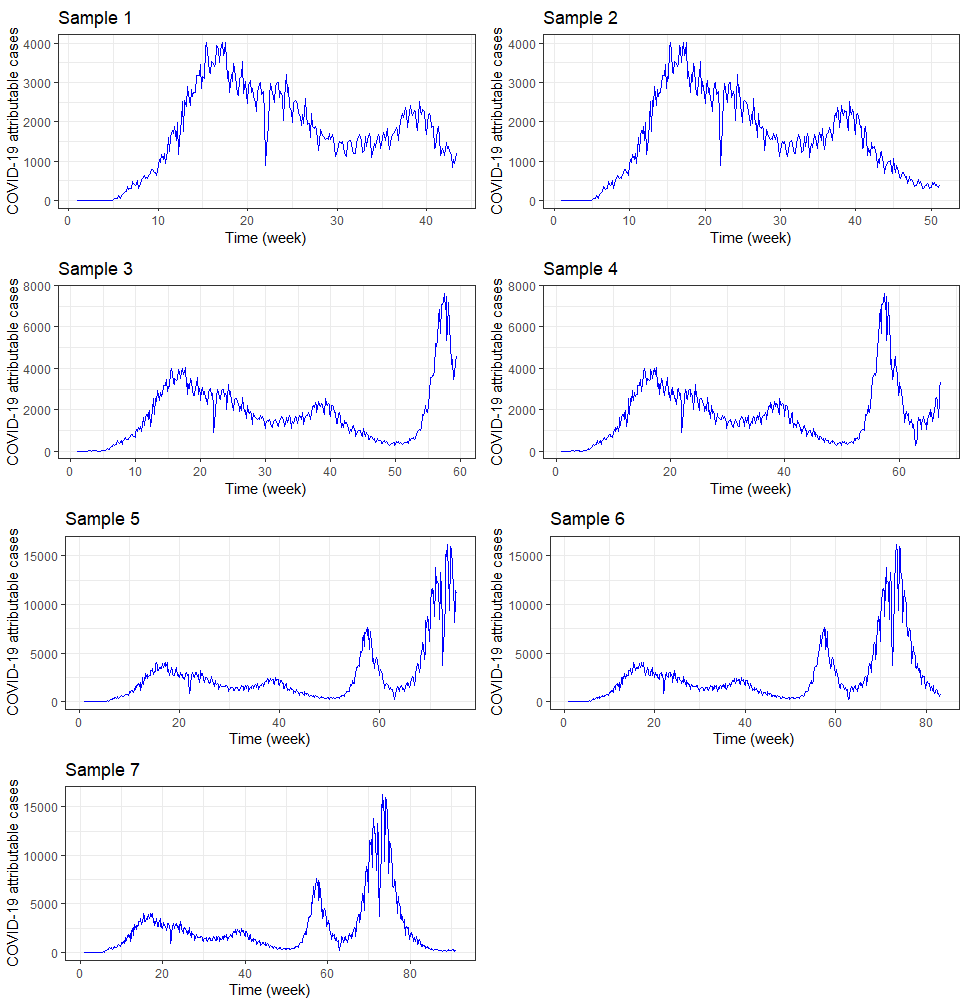


**Fig A. Time series plot of COVID-19 confirmed cases of the seven divided samples.**

**
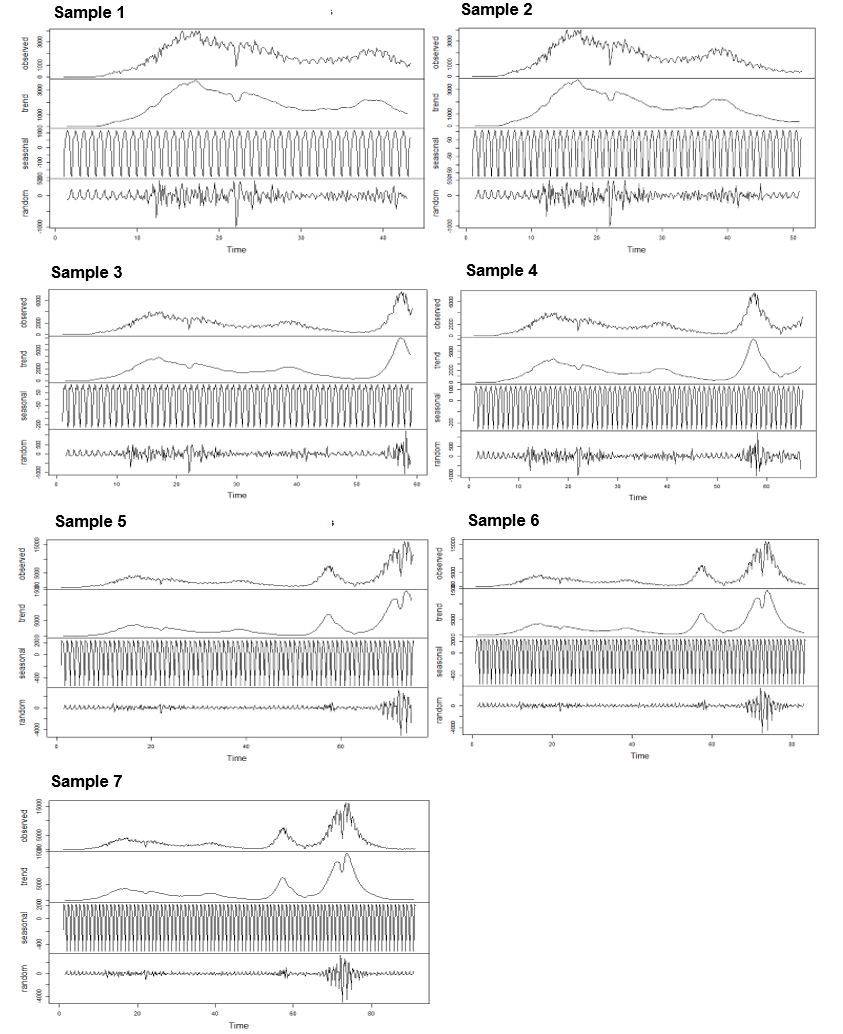
**

**Fig B. Seasonal decomposition of the Box-Cox transformed COVID-19 confirmed cases**


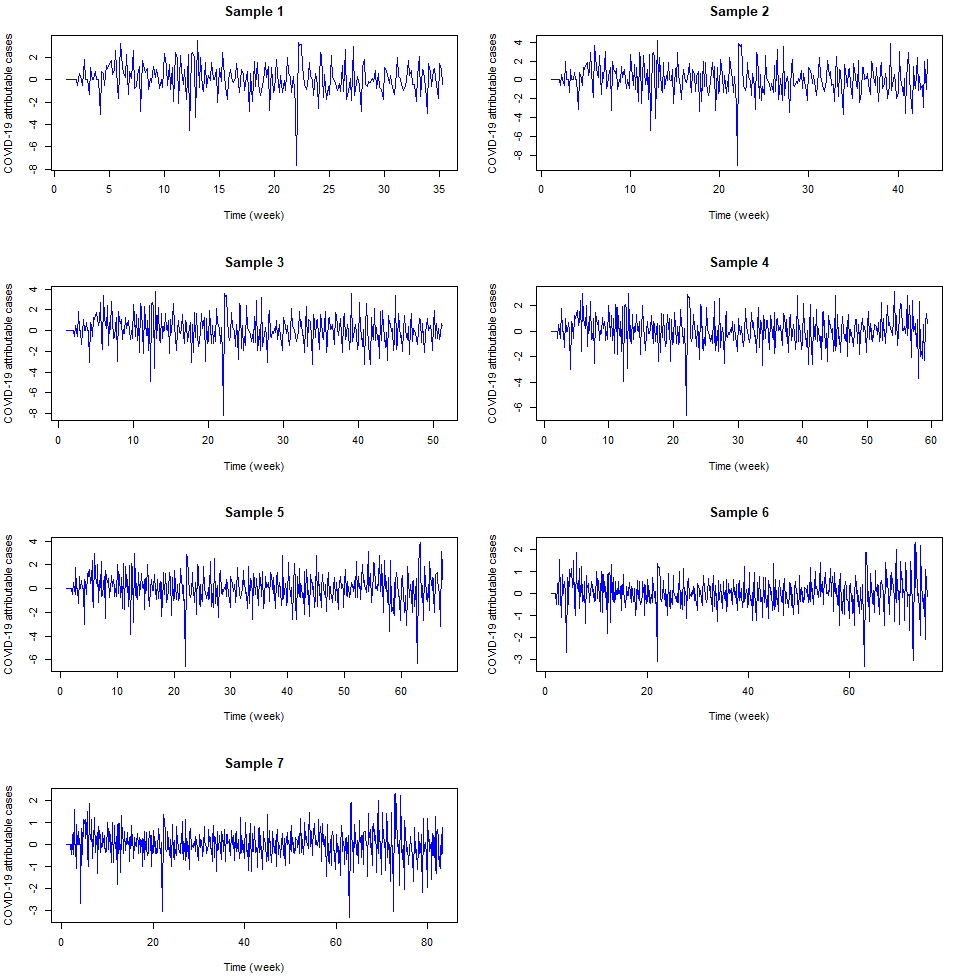


**Fig C. First order differencing of the training data sets for confirmed cases show stationarity.**

**Table A. Estimated parameters of the ARIMA model for different training sets for COVID-19 confirmed cases.**

| Training sets | ARIMA models | AR (1) | SAR (1) | SAR (2) | MA (1) | MA (2) | SMA (1) | SMA (2) |
| --- | --- | --- | --- | --- | --- | --- | --- | --- |
| Train 1 | ARIMA(0,1,1)×(0,1,1)_7_ |  |  |  | -0.35 |  | -0.80 |  |
| Train 2 | ARIMA(0,1,1)×(2,1,1)_7_ |  | 0.15 | 0.15 | -0.36 |  | -0.92 |  |
| Train 3 | ARIMA(0,1,2)×(2,1,1)_7_ |  | 0.16 | 0.15 | -0.36 | -0.06 | -0.93 |  |
| Train 4 | ARIMA(1,1,2)×(0,1,2)_7_ | 0.94 |  |  | -1.26 | 0.33 | -0.80 | -0.09 |
| Train 5 | ARIMA(0,1,1)×(2,1,2)_7_ |  | -0.09 | 0.21 | -0.19 |  | -0.70 | -0.22 |
| Train 6 | ARIMA(0,1,1)×(2,1,1)_7_ |  | 0.10 | 0.14 | -0.10 |  | -0.91 |  |
| Train 7 | ARIMA(0,1,1)×(2,1,1)_7_ |  | 0.11 | 0.16 | -0.12 |  | -0.93 |  |

ARIMA: Autoregressive Integrated Moving Average

The fitted ARIMA models were then used to predict the COVID-19 confirmed cases and the test data sets were used to evaluate the predictive performance of the models. The mean absolute error (MAE), root mean square error (RMSE), mean percentage error (MPE) and mean absolute percentage error (MAPE) were used to evaluate the models performances (main text Table 2).

The XGBoost model were built by frequently adjusting different parameter like seas_method= ‘dummies’, trend_method= ‘none’, power transformation parameter lambda for each training set. After that the built XGBoost model was used to predict the COVID-19 confirmed cases and the test sets were used to evaluate the models predictive performance. The evalution parameters of the model were shown in main text (Table 3).


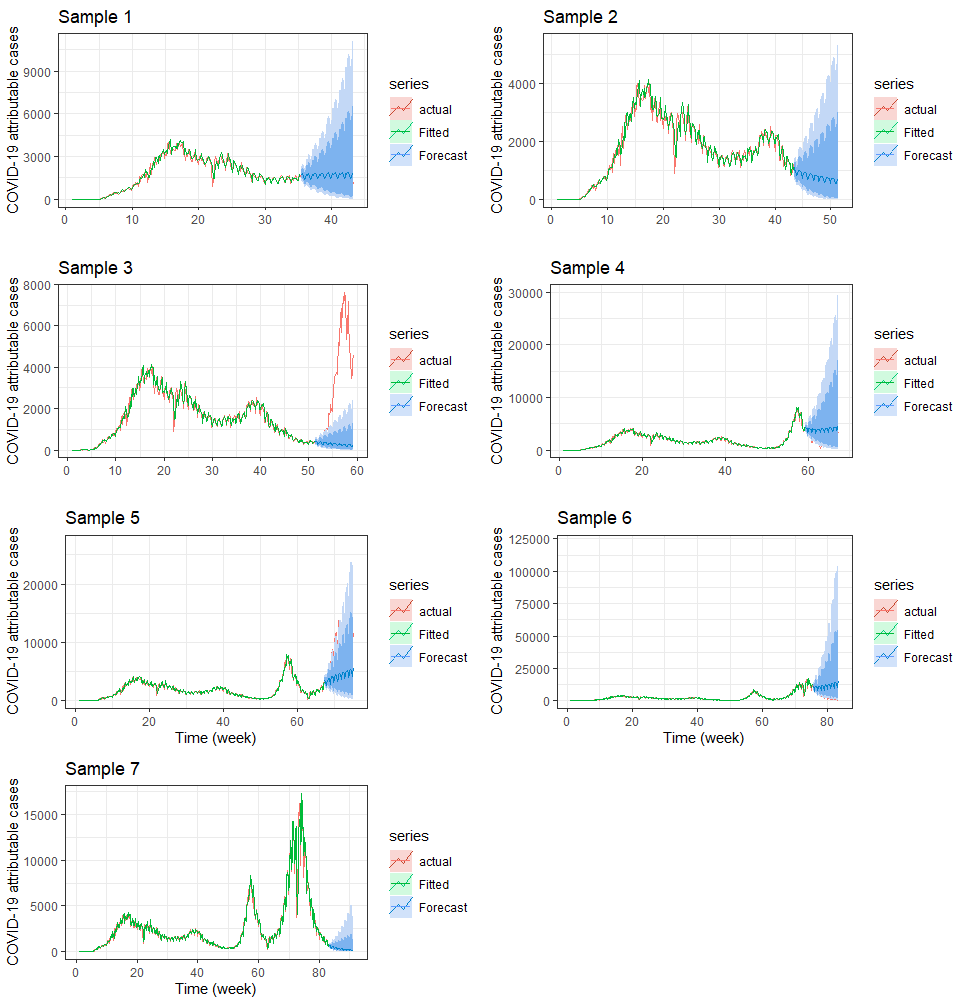


**FigD. Prediction plot of COVID-19 confirmed cases using ARIMA model.**


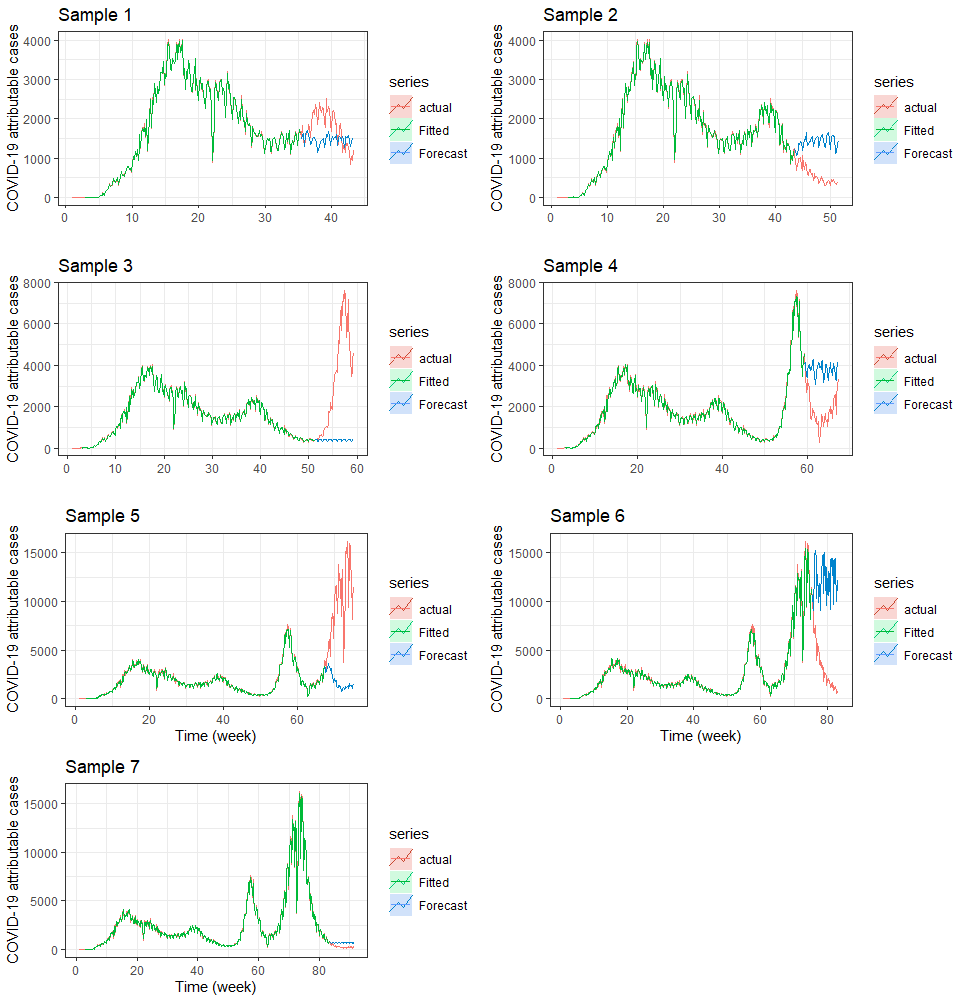


**Fig E. Prediction plot of COVID-19 confirmed cases using XGBoost model.**

For COVID-19 deaths, the entire data was divided into 5 training and 5 test sets. The COVID-19 deaths data showed nonlinear trend and weekly seasonality (Fig F). We then performed Box-Cox transformation to the COVID-19 deaths data to reduce the variance and make the raw data stable. The transformed data were then decomposed to find the seasonality pattern. The decomposed data showed weekly seasonality (Fig G). After first differencing, the data was found stationary (Fig H). Like COVID-19 confirmed cases, the ARIMA and XGBoost models for COVID-19 deaths were built following the same procedures. The estimated parameters of the ARIMA models for COVID-19 deaths were shown in Table B.


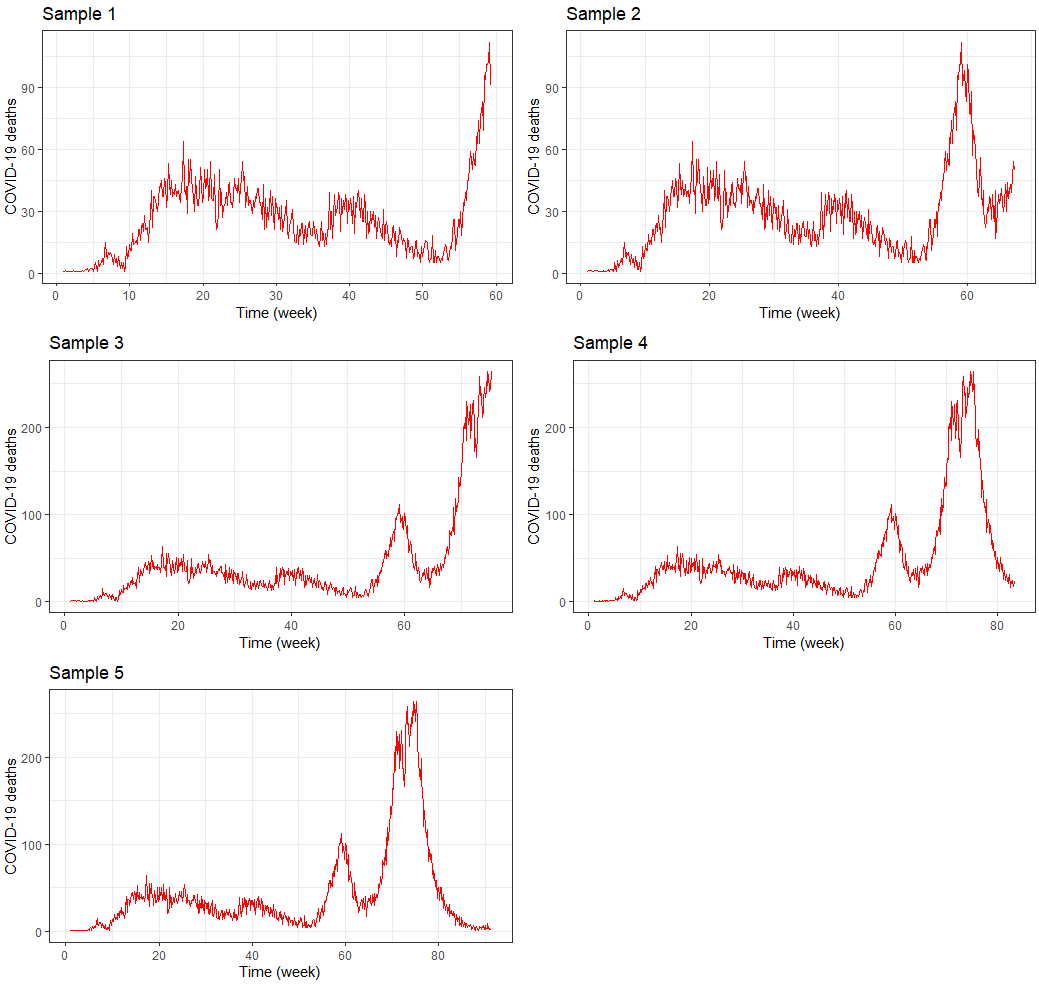


**Fig F. Time series plot of COVID-19 deaths of the five divided samples.**

**
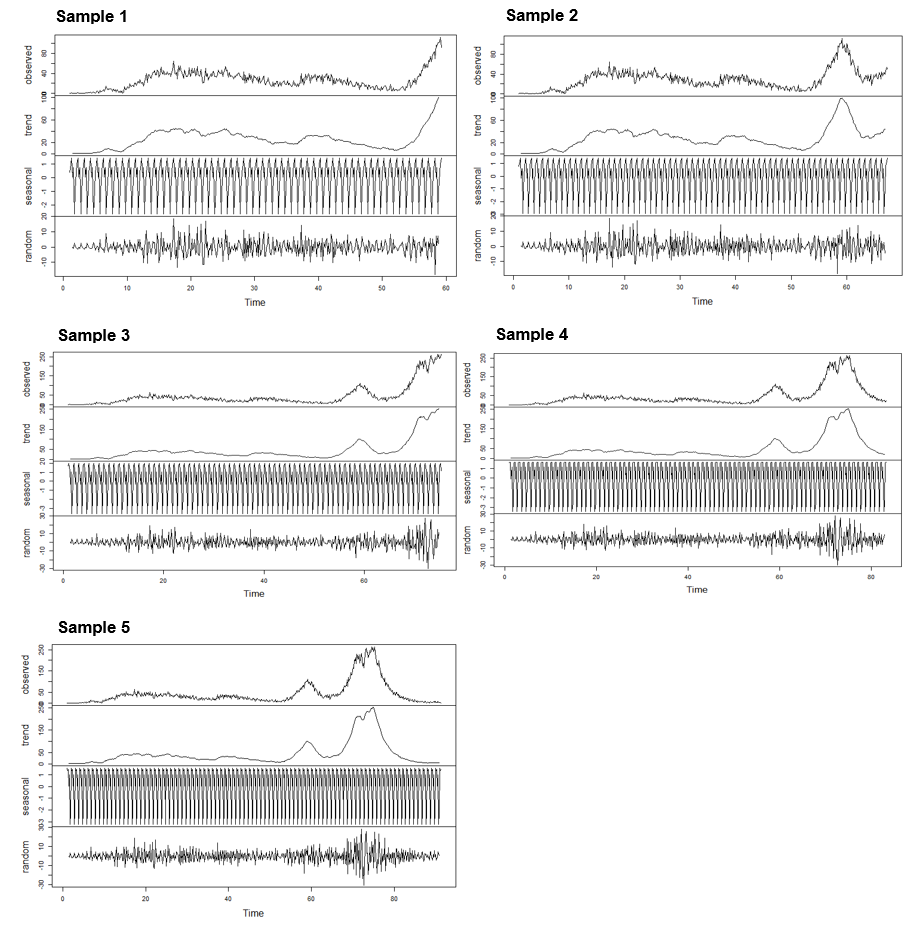
**

**Fig G. Seasonal decomposition of the Box-Cox transformed COVID-19 deaths**


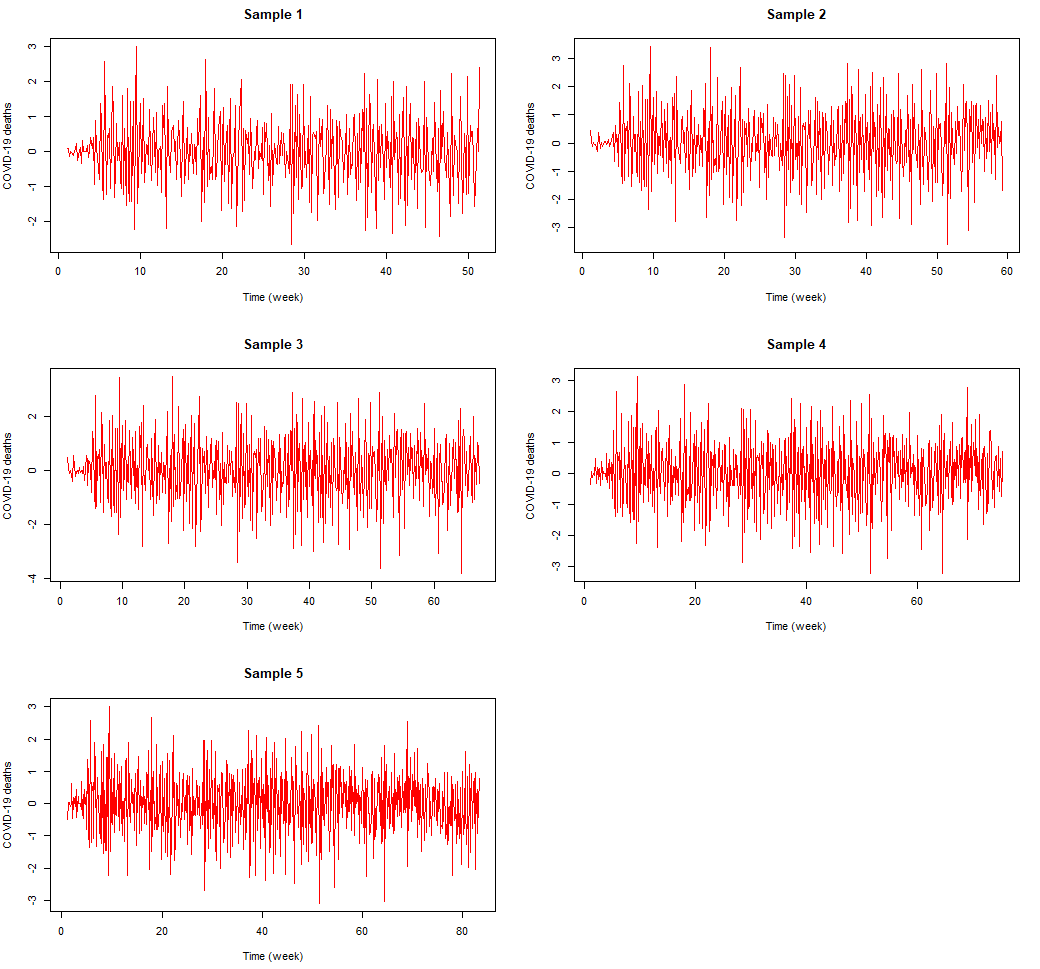


**Fig H. First order differencing of the training data sets for deaths show stationarity.**

**Table B. Estimated parameters of the ARIMA model for different training sets for COVID-19 deaths.**

| Training sets | ARIMA models | AR (1) | SAR (1) | SAR (2) | MA (1) | MA (2) | MA (3) | SMA (1) |
| --- | --- | --- | --- | --- | --- | --- | --- | --- |
| Train 1 | ARIMA(0,1,2)×(0,1,1)_7_ |  |  |  | -0.76 | 0.09 |  | -0.90 |
| Train 2 | ARIMA(1,1,2)×(0,1,1)_7_ | 0.80 |  |  | -1.53 | 0.64 |  | -0.92 |
| Train 3 | ARIMA(1,1,2)×(2,1,0)_7_ | 0.76 | -0.63 | -0.30 | -1.47 | 0.59 |  |  |
| Train 4 | ARIMA(1,1,2)×(2,1,0)_7_ | 0.77 | -0.62 | -0.29 | -1.44 | 0.56 |  |  |
| Train 5 | ARIMA(0,1,3)×(0,1,1)_7_ |  |  |  | -0.62 | 0.06 | 0.09 | -0.93 |

**
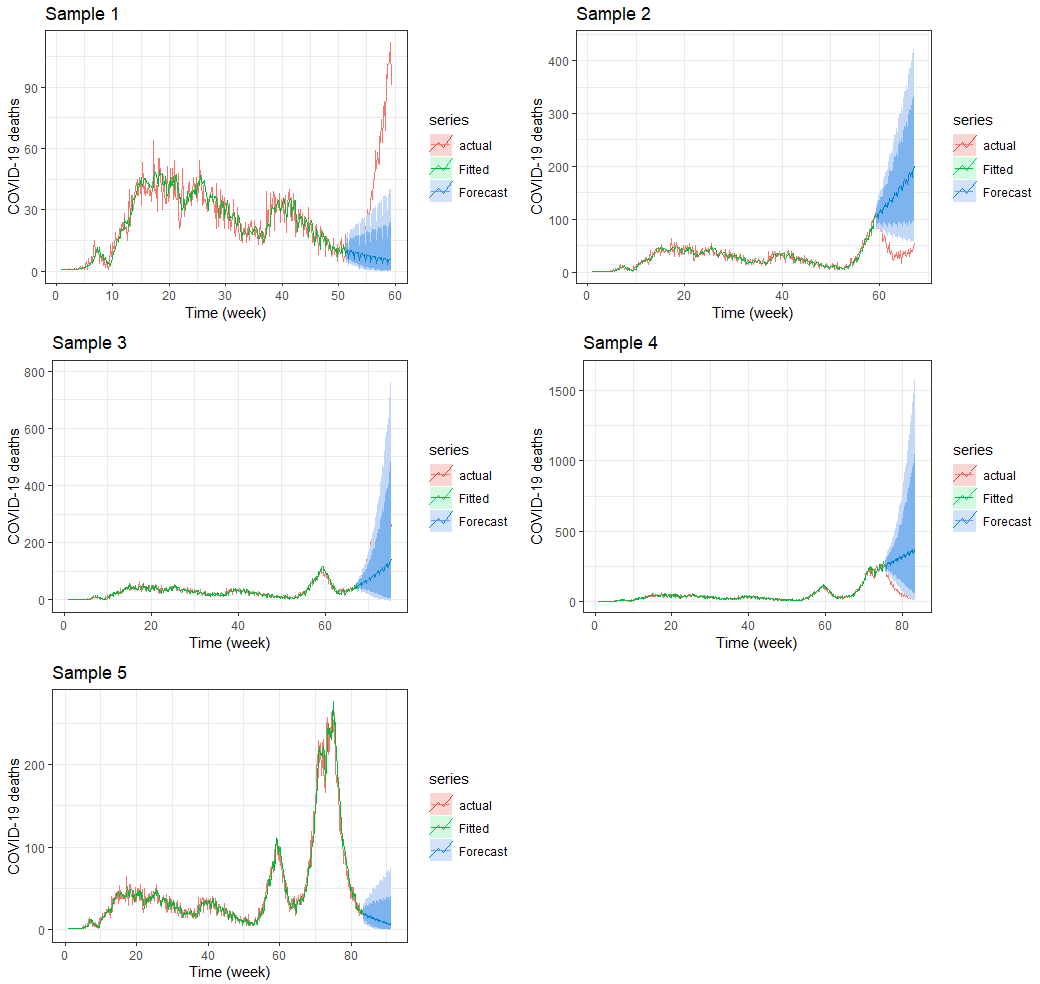
**

**Fig I. Prediction plot of COVID-19 deaths using ARIMA model.**

**
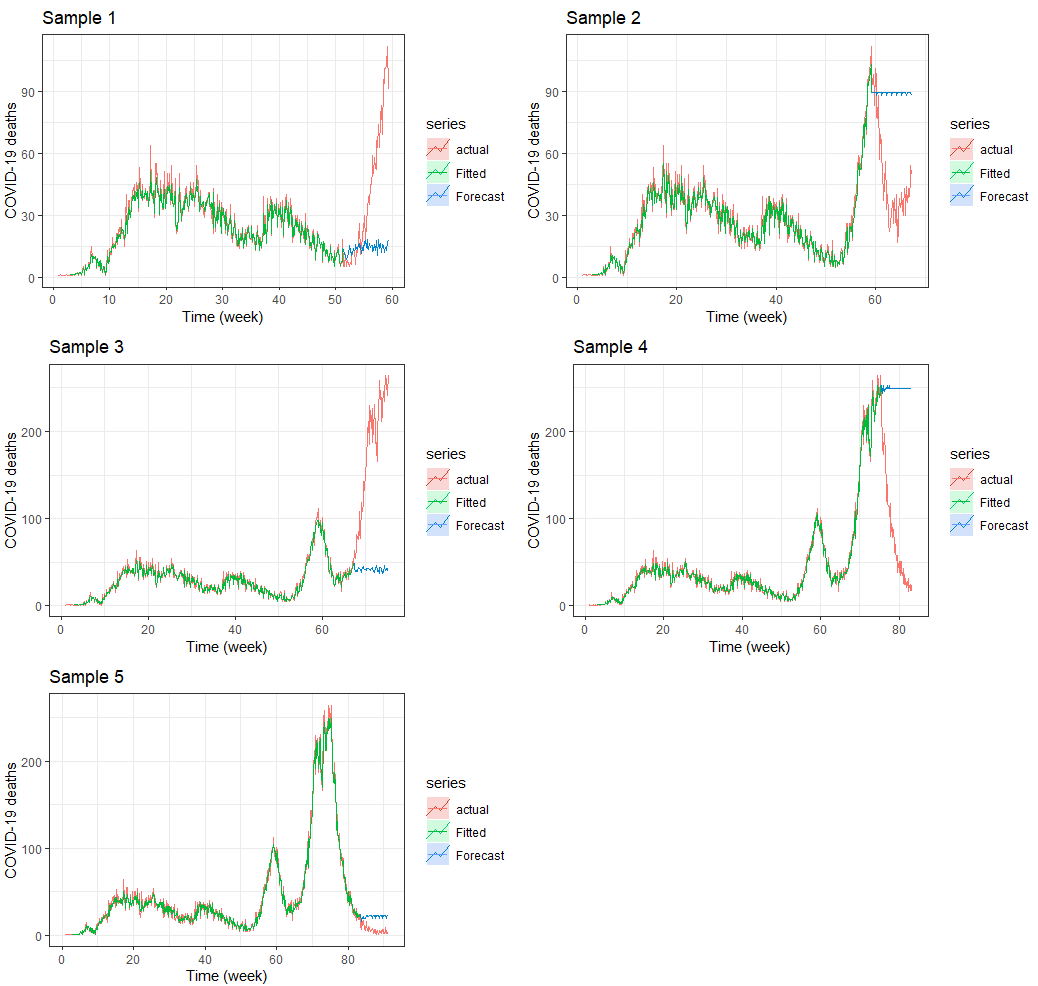
**

**Fig J. Prediction plot of COVID-19 deaths using XGBoost model.**

**References**

1. Curran-Everett D. Explorations in statistics: The log transformation. Adv Physiol Educ. 2018;42: 343–347. doi:10.1152/ADVAN.00018.2018

2. Rosselló J, Sansó A. Yearly, monthly and weekly seasonality of tourism demand: A decomposition analysis. Tour Manag. 2017;60: 379–389. doi:10.1016/j.tourman.2016.12.019
