# Supplementary material for: Accuracy comparison of ARIMA and XGBoost forecasting models in predicting the incidence of COVID-19 in Bangladesh
Source: PLOS Glob Public Health. 2022 May 18;2(5):e0000495. doi: 10.1371/journal.pgph.0000495 (PMC10021465; doi:10.1371/journal.pgph.0000495)
Supplement: Attachment — Submitted filename: Revision-notes.docx [file pgph.0000495.s003.docx]

**PLOS Global Public Health**

Accuracy comparison of ARIMA and XGBoost forecasting models in predicting the incidence of COVID-19 in Bangladesh

Manuscript Number: PGPH-D-21-00586

**Reviewer’s response**

Reviewer #1: Review of “Accuracy comparison of ARIMA and XGBoost forecasting models in predicting the incidence of COVID-19 in Bangladesh”

Thank you for the opportunity to review this manuscript. I found it interesting and enjoyed reading it. I think work assessing the predictive performance of COVID-19 forecasting models is needed, especially as we are now starting to reflect on what has gone well/poorly in this pandemic management. I do have some concerns about the overall vision for this analysis, as well as a few key analytical decisions. I think this work is not publishable in its current form, but has potential if the authors wish to undertake a considerable amount of work to provide new analyses and revisions to the framing of the text.
**Major Comments:**
1. My biggest concern is that it seems problematic to me that a single holdout period was chosen. It has been shown that predictive performance massive varies by time period, even for the same model using the same methods. Models do not perform equally during the various sets of circumstances that can arise (trends decreasing/increasing, speed of change, overall shape, etc.). I would recommend running this analysis on a set of training/test sets, iterating over all possible cutoff dates, and presenting aggregated predictive performance results, perhaps also stratified by month or season.
**Response**: Thanks for your suggestion. We reanalyzed the data and we also splitted the dataset into training and test set (please see lines 130-135, and table 2 and table 3) accordingly.
2. It is also not clear to me why a 1 week testing set was used. Longer-range forecasts are likely much more useful for policymakers. I would recommend seeing how these models perform at least 4 weeks out, but arguably even much longer range forecasts, like 8 to 12 seeks, are much more actionable from a policy perspective.

**Response**: We have extended the time of test dataset up to 8 weeks (please see lines 134-135 and Supplementary-file-3 and table S1 and table S2).

3. Why were cases chosen and not deaths as the outcome measure of interest? Both are flawed measures depending on health system and surveillance factors, but deaths are generally much more reliable. Cases can swing wildly based on shifts in testing rates, etc.

**Response**: We have made prediction of both confirmed cases and deaths in our new analysis (Please see table 3 and table 4).

4. I would recommend also assessing median error or median percent error, a measure of bias, alongside measures of accuracy.

**Response**: Thanks for your suggestion. We assessed and included median percentage error measure of accuracy (Please see table 3 and table 4).

5. Overall, I felt there was a lack of interpretation of the implications of the results for the real world. One model was found to have a better predictive performance. But more perspective is needed on why that is consequential. Thinking through why this work matters, and for whom, can also help sharpen the analytical choices.

**Response**: We have revised the discussion section section and discussed the above issue (Please see lines 361-367, 396-400, 407-417).

6. For example, why where these models compared only on data from Bangladesh. If this paper is about these methods, then comparing them on data from many more countries would provide a much more robust analysis (and it does not take much more work to compare 1 country or 100, given that these analyses are conducted in R). But if this paper is about the unique context of Bangladesh, and providing information for policymakers from that country, then that needs to be clearer.

**Response**: We performed these models on the COVID-19 data of Nepal (please see Supplementary-file-3 and also see lines 413-417).

7. I think there is too much emphasis on the in-sample predictive performance of these models, which isn’t necessarily important for a forecasting model.
**Response**: The study aimed to develop a suitatble model that will best predict the COVID-19 confirmed cases and deaths in Bangladesh. We made a comparison between two models accuracy to predict in which we emphasized on the in-sample predictive performance for developing the suitable model.

8. Data and code availability is not specified in the piece (that I can see) which would be a violation of journal requirements for publication.
**Response**: We have included the data (Supplementary-file-1) and codes (Supplementary file-2), and Github also (please see reference 50).

Minor Comments:
1. In the figures, the time scale makes it difficult to see the actual predictions vs. subsequently observed reality. I would recommend showing fewer data points to make the predictions more legible. Especially since the im-sample fit doesn’t really matter for the main goals of this paper, unless I am misunderstanding them.
**Response**: We edited the figures accordingly (please see the Fig 7, Fig 8).

2. Some of the details about how models were fit (e.g. auto.arima() used in R) were not available in methods, where I expected them, but rather the results section, which made me initially think they were missing

**Response**: We have mentioned the issue in the method section (please see lines 230-231).

Reviewer #2: This study looks at the COVID-19 epidemic in Bangladesh. COVID-19 cases from March 2020 to August 2021 were retrieved and divided into a training [March 2020-August 19 2021] and test [August 20 2021, August 26 2021] sets. The authors fit the data with an autoregressive integrated moving average (ARIMA) and a regularised gradient boosting (XGBoost) models on the training set and compare their prediction performances on the test set. Authors conclude that the ARIMA is not flexible enough to fit well the data while the XGBoost obtains encouraging prediction performance.

**MAJOR COMMENTS**
- My biggest concern about this paper as it currently stands is the lack of a clear link making it of direct relevance to public health. While the authors explain the catastrophic consequences of COVID-19 on Bangladesh’s economy, education and health care, they do not demonstrate how predictions from their models regarding confirmed cases could have been used to tackle the crisis or provide useful actionable information to policymakers. Further explanation and discussion (both of this and the limitations of their approach) is therefore required.
**Response**: We included the explanation (Please see lines 102-109, 443-452)

- A second issue is that confirmed cases are not easily interpretable - they depend on many time-varying factors (such as testing availability, healthcare seeking behaviours etc). It is unclear then whether fitting these models to case data is picking up legitimate epidemiological signals. It is this reason that has motivated many epidemiological models to use COVID-19 deaths as an indicator of the progression and trajectory of SARS-CoV-2 epidemics (see e.g. Flaxman et al., Nature). The authors might consider fitting their models to deaths instead of case data, though note (see below) that irrespective of the underlying dataset used, I also have significant concerns about the statistical approach adopted.
**Response**: Thanks for your advice. We reanalyzed the data and also made prediction of deaths in the revised manuscript (Please see table 2, table 3 and table 4).

- The time-period spanned by the test dataset (August 20th to 26th 2021) is very small compared to the period spanned by the training dataset (March 1st 2020 to August 19th 2021). Epidemiological quantities (such as the reproduction number) driving the dynamics of the case counts are unlikely to fluctuate substantially over such short time-periods – it is therefore unclear whether the models are successfully capturing the underlying epidemiological phenomena driving temporal fluctuations in case counts. It is necessary to explore a range of training and test data set sizes, and particularly, for the authors to evaluate model performance over a time period where the reproduction number clearly changes (e.g. weeks 45-55 or 60-70 for example where the declining case curve then goes back up again).
**Response**: We have extended the time of the test dataset (please see lines 134-135 and Supplementary-file-3, table S1 and table S2).
- There is currently a lack of explanation and description of the chosen statistical methods (and the inference approach i.e. the algorithms used to fit the model). Whilst models similar to XGBoost have been used for time-series forecasting, this is typically done by first generating/collating various features potentially relevant to the dataset (such as day-of-the-week, seasonality etc) and then training the model using these features. It is not clear currently what the authors did as the statistical explanations are too brief and not specific enough. Moreover, if the authors did not use this feature-based approach and instead trained it directly on the time-series data itself, I have significant concerns as to the validity of that choice (I am not aware of other published examples of approaches like this and am unsure whether such an approach is valid).
**Response**: We revised the method section and provided the necessary explanation and description of the chosen statistical methods (please see lines 139-142, 179-183).

- The authors claim that XGBoost has good prediction accuracy – description of this is quite limited currently however, and only indirectly quantified through comparison to ARIMA.
**Response**: We reanalyzed and discussed the above issue in the discussion section (please see table 2, table 3, table 4 and and lines 407-413).


DETAILED COMMENTS
ARIMA:
- Equation (1): why the ‘y’ on the LHS is in capital and not the ‘y’s on the RHS?

**Response**: We made correction in equation (1).

- I do not follow how the model is defined. The authors state that the model is decomposed in two parts, a ‘simple seasonal model’ (in eq 4) and a ‘product seasonal model’. Where is eq.3 used? And how are S_t, T_t and I_t found?
**Response**: Equation (3) has been written to describe the ARIMA model. Plotting the data we found the weekly seasonality information in the data (S_t), trend information.
Gradient boosting model:
- Gradient boosting has been shown to be efficient in cross-sectional data. The authors lack to reference/demonstrate why using this method on time series makes sense theoretically.

**Response**: We used XGBoost regressor for time series prediction. We included the reference (please see reference number 41).

- “The model may need to be iterated multiple times or more”: the authors must define what iterated means in this context.

**Response**: We reanalyzed regarding the above issue (please see lines 189-190).

- ‘cost function’ à ‘loss function’

**Response**: We edited accordingly (please see lines 196).

- Equation (5): What is x_i? Similarly in this equation and all that apply, I understand what y_i means from context but I do not think it is defined anywhere.

**Response**: We made corrections to the equation and included the details (please see line 195).

- Equation (5): It is hard to follow this equation, f_m has input x_i in one instance and not the next, and what is the operation underlined in (y_i, y_i^{m-1})? Lastly the loss function has one input and not two? Do the authors meant L[y_i, (y_i^{m-1} + f_m(x_i))]?

**Response**: We revised and made corrections on the equation (please see line 194)

- What are the covariates (or features) used to fit this model? The week indices?

**Response**: We performed univariate time series modeling and so no covariates were present here (please see lines 443-452).

- The authors do not provide sufficient detail about the way in which parameters of the gradient boosting model were optimised – if fixed parameters were eventually chosen, that is fine, but it should be clearly stated. If hyperparameter optimisation was undertaken, then an explanation about how this was carried out is required.

**Response**: We didn’t optimize hyperparameter. We included the details about the parameters (please see lines 314-318).

Evaluation parameter of models:
- This section states elementary statistics and could be moved to the supplements or removed.
- Why do the authors desire to compare three different metrics? Why not pick only one?
**Response**: The mean absolute error (MAE), root mean square error (RMSE), and mean absolute percentage error (MAPE) are the most widely used measures of accuracy. This is the reason we have used these three measures in the study. We kept the formula in the manuscript.
Application ARIMA:

- The motivation for the Box-Cox transform is not clear “to make the dataset more stable”. What is the definition of stable in this context? Especially because The Box-Cox transform creates two huge drops at the beginning of the sequence. And the large increase at the end of the sequence is now almost at the same magnitude as the first wave. Could the authors provide a sensitivity analysis where the original data are fitted? Are the original data just tweaked to fit the assumptions of the ARIMA?

**Response**: We have discussed the above issues (please see lines 253-256).
- What is the cause of the seasonal effects according to the authors? They briefly discuss environmental effects in the discussion without more explanation on their relation to confirmed cases. Could it be that there is a drop in the week-ends because less health care workers are employed to report the new cases/less people get tested, and the spike at the beginning of the week to account for reporting delays? (underlying again the issues arising from using confirmed cases data)

**Response**: Please see the discussion section (please see lines 356-357).

- What is the difference between the AIC and the AICc? The authors mention both

**Response**: We have adjusted the issue (please see lines 289, 295).

- Why the AIC is used for model selection of the ARIMA and other metrics for model comparison between the ARIMA and gradient boosting?

**Response**: AIC represents Akaike's information criterion used to compare between/among two or more ARIMA models to find the best fitted ARIMA model so we used it. For comparison between/among different models, we used three different measures of accuracy (MAE, RMSE and MAPE) for choosing the best model.

- ‘The arima model was built with the auto.’ Is this sentence not finished somehow?

**Response**: We corrected the above issue (please see lines 287-288).

Application Gradient boosting:
- ‘The XGBoost model with the greatest results was developed by changing the parameters frequently’, the authors should clarify what this means in context, do they fit the model weekly or monthly?
**Response**: We reanalyzed the data (please see line 313, table 2, table 3 and table 4)
Prediction:
The prediction part is not convincing. The authors fit the entire data set up to the before to last week and only predict the confirmed number of cases in the last week.

- Figure 7 and 8 do not convince me of the efficacy of any of their methods for prediction as their point estimates are off.

**Response**: We included the point estimates (please see the Supplementary-file-3, table S1 and table S2).

- Even more concerning is the prediction by the ARIMA of a negative number of cases at the beginning of the sequence.

**Response**: Please see the Fig 7 and Fig 8.

- Do the authors have an explanation as to why the XGBoost method does not manage to predict well the spikes included the training set towards the end of the data set?

**Response**: We reanalyzed the data (please see lines 407-413, table 3, table 4).

- Could the authors show the confidence intervals of their predictions?

**Response**: Please see Supplementary-file-3 (table S1).

- Could the authors show multiple prediction scenarios where more days are predicted?

**Response**: Please the Supplementary-file-3 (table S1 and table S2).

- Most importantly theoretically, the authors lack to demonstrate how the information collected in the first weeks inform the predictions at week 70+? (1) Up to a certain point, the information of the past has no impact on the future, and therefore most of the dataset could be excluded and (2) what epidemiological signal is learned.
**Response**: Please see lines 360-362.

Discussion:
- The authors argue for a beneficial effect of the ‘autoregressive and average moving models’. A more thorough discussion of those benefits is necessary.

**Response**: We revised the discussion section (please see lines 361-367).

- “the training and test sets performed very differently”, a data set does not perform.

**Response**: please see the table 2, table 3 and table 4.

- The evidence for calling gradient boosting’s performance ‘outstanding’ are not strong. In order to make this claim, the predictions obtained with gradient boosting should be compared to more methods that could in theory perform well (i.e., not ARIMA) and previous studies that tackled the same problem.
**Response**: We reanalyzed the data and discussed accordimgly the issue (please see table 2, table 3 and table 4, and lines 407-413)
Figures

- Informative axes title (e.g., ‘Confirmed COVID-19 attributable cases’ instead of ‘cases’ for Figure 1) would be appreciated

**Response**: We changed the title (please see the Fig 1).

- Dating the y axis would be more informative than the week indexing.

**Response**: We did week indexing to show the weekly simpact.

- Figure 6 does not have a y-axis title on the top plot

**Response**: We added the y-axis on the top plot of figure 6.

- Labelling plots by A, B, C in Figure 6 would make the explanation of the figure clearer.

**Response**: We labelled the plots accordingly (please see the Fig 6).

- Self explanatory caption and columns label for table 1 would be useful
**Response**: We edited the table (please see the table 2).

Typos:
- I spotted some ‘covid-19’ instead of ‘COVID-19’

**Response**: We edited accordingly whereever we found any typos (Please see lines 39).

- Some ‘arima’ instead of ‘ARIMA’

**Response**: Please see lines 287.

- Some sentences would benefit polishing, e.g., ‘the number of cases is increasing significantly after 400 days from the beginning in the country’.

**Response**: We made corrections (please see lines 349-354).

- Some terms should be chosen more rigorously, e.g., ‘fitting and prediction impact’
**Response**: We incorporated the above suggestion (Please see line 408).

References
Flaxman, S., Mishra, S., Gandy, A. et al. Estimating the effects of non-pharmaceutical interventions on COVID-19 in Europe. Nature 584, 257–261 (2020). <https://doi.org/10.1038/s41586-020-2405-7>
