# Supplementary material for: Accuracy comparison of ARIMA and XGBoost forecasting models in predicting the incidence of COVID-19 in Bangladesh
Source: PLOS Glob Public Health. 2022 May 18;2(5):e0000495. doi: 10.1371/journal.pgph.0000495 (PMC10021465; doi:10.1371/journal.pgph.0000495)
Supplement: Attachment — Submitted filename: Response to Reviewers.docx [file pgph.0000495.s004.docx]

PLOS Global Public Health

PGPH-D-21-00586R1
Accuracy comparison of ARIMA and XGBoost forecasting models in predicting the incidence of COVID-19 in Bangladesh

Reviewer #1: The authors have undertaken considerable work to update the analyses, which are greatly appreciated, and have strengthened the research. However, the work still uses a single training and a single testing period. Looking at Figures 7 and 8 it become clear why this is not appropriate. The forecasts are assessed relative to only a tiny window of the overall pandemic, and at a time when rates where monotonically decreasing. These results will likely not be generalizable to the remainder of the timeseries where deaths are rising or peaking. Instead, for this work to be robust, the authors must average predictive performance over a series of testing and training holdouts. This would entail iterating over all possible start dates (e.g. each week from May 2020 to October 2021), fitting the model on all available data, and checking predictive performance on the remainder of the timeseries. Predictive validity statistics can then be averages across all the various testing windows.

**Comment:** We applied the method cross-validation on our time series data. The approach begins with a small subset of data for training, forecasts for future data points, and then verify the accuracy of the predicted data points. Following that, the same predicted data points are incorporated in the next training dataset, and more data points are predicted. We also divided the data into a series of testing and training holdouts and averaged the predictive performances for each model as you suggested (please see lines 300-302 and supplementary file 4).

All other edits made by the authors are satisfactory.

**Comment: Thank you.**

Reviewer #2: The manuscript has been improved most notably by the inclusion of the predictions of confirmed covid-19 deaths. I also remarked an improvement of the writing and the motivations for predicting covid-19 deaths. And I commend the authors for both. That being said, the paper still falls short, in my opinion, in a clear explanation of the methods and the algorithms. The maths of the methods is not clear or incorrect, and the algorithm used for fitting is not stated. Saying that one used a specific package is not enough in this context. Some comments were only partly addressed or entirely ignored.

**Comment:** We made corrections on the algorithms and equations we stated (please see lines 144, 148-149, 158-166). We also explored a range of training and test sets of the dataset, fitted the models and finally averaged the predictive accuracy of each model for both confirmed cases and deaths (please see supplementary file 4).

The conclusions of the authors did change between the previous version and the revision. I believe due to the extension of the test period. ARIMA is now found to perform better. The fact that the gradient boosting method does not perform better is not problematic for me. Even bad results are important for science to progress. However, gradient boosting must be fed with information (in the same way that the ARIMA has a specific parametric form which induces seasonal trends). And it is still not clear to me what information (i.e., features) have been given to the gradient boosting. In this context I cannot be convinced of the poor performance of gradient boosting.
**Comment:** We used week indices as input features. We provided the seasonality, trend information and the power transformation (Box-Cox) information to the build XGBoost model (please see lines 264-267 and 351-356). We also added some additional analysis to check the accuracy of our proposed model in supplementary file 4).

ARIMA MODEL

-Are X_t covariates of the seasonal model?

**Comment:** Yes.

-To my best knowledge, the authors did not explain what P and Q were in l.153-154 and the authors did not state what d from ARIMA(p, d, q) governed and how it was estimated.

**Comment:** We explained the aforementioned term in the revised manuscript (please see lines 134-135 & 163-164)

-In the response to reviewers, the authors did not specify rigorously how to find S_t, T_t and I_t
-Eq (2) and (3) it should be theta_q not theta_p.

**Comment:** We included the explanation (please see lines 158-166 and reference no. 42). We have made corrections on equations (2) and (3).

XBOOST
- A Loss function in (5) is defined by two outputs and may be written as L(y, ybar), which is still not the case.

**Comment:** We added a reference (please see reference 42).

“- What are the covariates (or features) used to fit this model? The week indices?”. Their answer “no covariates were present here” However in the text they say “x_i is the feature vector”. So again, what are the features?

**Comment:** We used week indices as features to fit the XGBoost model.
