# Supplementary material for: Accuracy comparison of ARIMA and XGBoost forecasting models in predicting the incidence of COVID-19 in Bangladesh
Source: PLOS Glob Public Health. 2022 May 18;2(5):e0000495. doi: 10.1371/journal.pgph.0000495 (PMC10021465; doi:10.1371/journal.pgph.0000495)
Supplement: Attachment — Submitted filename: Response to Reviewers.docx [file pgph.0000495.s005.docx]

PLOS Global Public Health

PGPH-D-21-00586R1
**Accuracy comparison of ARIMA and XGBoost forecasting models in predicting the incidence of COVID-19 in Bangladesh**

Reviewer #1: I appreciate the author's efforts to revise the piece. They have now provided a supplemental analysis using several different training and testing sets. However they have not changed the results in the main text which still rely on a single testing/training split. I would argue that the results shown in the main text (which were not revised on the last round, according to the tracked changes document) need to show the average across the various training/testing splits. Taking a look at their R code it appears to only run a single train/test split at a time. The reasons why this is insufficient were explained in my last round of review and are clear in the literature. Unless the authors can update the main text results to reflect the out-of-sample predictive performance averaged across a wide range of training/testing splits, I cannot support this work being published, because the results will simply be representative of one minor moment in a much larger pandemic.

**Comment:** We updated the result in the main text where the average across the various training/testing splits was shown (please see lines 48-61, Table 2, Table 3, Table 4 and Table 5).

Reviewer #2: The paper has been improved with the addition of new prediction analyses. However, in my opinion, the paper still requires much attention specifically in the methods section.

- The authors have made minor and insufficient changes regarding my previous comments on the need for clearer explanation on the different parts of the ARIMA model.

**Comment:** Based on your previous comment, we have now added the explanation to specify the different parts of the seasonal ARIMA model (please see lines 175-190).

- Equation (3): shouldn’t there be only one epsilon_t?

**Comment:** We have made corrections in equation 3 (please see lines 163-164).

- The authors STILL have not fixed the loss function. FYI reference [42] has a loss function which is formulated as l(x,y) (eq.6 in ref [42]).

**Comment:** We edited the loss function accordingly (please see lines 206-207).

- The authors stated that the only covariate of the XGBoost were the week indices. It is therefore clear why GBoost doesn’t perform. On the one hand you have a heavily parametrised ARIMA model, that harnesses information from seasonal and autoregressive trends, and on the other hand you have a powerful GBoost that is fed with no information. How would the GBoost has any predictive performance using only week indices?

**Comment:** We performed univariate time series modelling where the only covariate was the time (week). We adjusted different parameters of the XGBoost to get the best result and mentioned the limitation of our model’s poor performance in the limitation section (please see lines 479-486).

- The aim stated by this paper is already problematic. Because no model can make accurate prediction without incorporating epidemiological signals. So, the real aim of this paper is to compare, under partial information (i.e., here only the seasonal trends), which of the ARIMA or GBoost is capable of making the ‘best’ prediction (relative to the two models, not relative to models incorporating epidemiological trends proposed in the literature).

In this context, (1) in my opinion the authors should rephrase the aim in the abstract and introduction to clearly express the above point.

**Comment:** We rephrased the aim in the abstract and introduction (please see lines 34-39 and 115-121).

And (2) in order to make a fair comparison, it is essential that the authors find a way to give the same level of information to the two models. This could be achieved by using as covariate features potentially relevant to the dataset (such as day-of-the-week, seasonality etc). They can refer to their own citation such as [46].

**Comment:** We have updated analysis and result section providing same level of information to the two models. We divided the data (COVID-19 confirmed cases and deaths) into several training and test sets and averaged their predictive performances to make the comparison between ARIMA and XGBoost model (please see Table 2, Table 3, Table 4, Table 5 and Supplementary file 3)

I would like to finish my review by saying that all points above have been raised since the first round of reviews.
